# Differential role of insulin resistance and β-cell function in the development of prediabetes and diabetes in middle-aged and elderly Chinese population

**DOI:** 10.1186/s13098-019-0418-x

**Published:** 2019-03-05

**Authors:** Xueli Cai, Lili Xia, Yuesong Pan, Dian He, Huiping Zhu, Tiemin Wei, Yan He

**Affiliations:** 1 Department of Neurology, Lishui Municipal Central Hospital, Lishui, Zhejiang China; 2 Editorial Office of Hepatobiliary and Pancreatic Diseases International, the First Affiliated Hospital, College of Medicine, Zhejiang University, Hangzhou, China; 3 Department of Neurology, Beijing Tiantan Hospital, Capital Medical University, Beijing, China; 4 Department of Epidemiology and Biostatics, School of Public Health, Capital Medical University, Beijing, China

**Keywords:** Adipose tissue insulin resistance, Peripheral tissue insulin resistance, β-Cell function, Prediabetes, Type 2 diabetes

## Abstract

**Background:**

The prevalence of diabetes and prediabetes were estimated to be 10.9% and 35.7% in the Chinese adult population, respectively, and the middle-aged and elderly Chinese are at even higher risk of diabetes and prediabetes than younger population. With the increasing trend of aging in China, the burden of diabetes and related complications will be aggravated.

**Objectives:**

Through comparing the indices of insulin resistance and β-cell function between subjects with different glucose metabolic status, to analyze the differential role of insulin resistance and β-cell function in the development of prediabetes and type 2 diabetes (T2DM) in the middle-aged and elderly Chinese population.

**Methods:**

In this cross-sectional study, we enrolled 512 participants aged 50 and over. The indices of insulin resistance (homoeostasis model assessment of insulin resistance (HOMA-IR) and adipose tissue insulin resistance (Adipo-IR), and indices of β-cell function [HOMA-β), fasting C-peptide to glucose ratio (FCPRI) and postprandial C-peptide to glucose ratio (PCPRI)] were calculated. Association of insulin resistance and β-cell function with prediabetes or T2DM were evaluated by multivariate logistic regression analysis, in which potential confounding factors were adjusted.

**Results:**

Of the 509 participants with complete information, 263 (51.7%) had normal glucose tolerance (NGT), 161 (31.6%) were in prediabetic status and 85 (16.7%) were overt T2DM. With the advancing of unfavorable glucose metabolism, the insulin resistance (HOMA-IR and Adipo-IR) and β-cell function (FCPRI, PCPRI) deteriorated (*P*_trend_ < 0.05 for all indices). We found that increase in insulin resistance expressed by Adipo-IR and HOMA-IR is associated with increased risk of prediabetes, whereas decrease in β-cell function expressed by HOMA-β and PCPRI is associated with increased risk of T2DM. We also demonstrated that Adipo-IR was more closely associated with developing prediabetes than HOMA-IR, and PCPRI was most closely related with developing T2DM among the indices of β-cell function used in this study.

**Conclusions:**

Insulin resistance is the main determinant of developing prediabetes, whereas β-cell function is the main determinant of developing T2DM.

## Introduction

According to the recent nationwide survey of Chinese adult population, the prevalence of diabetes and prediabetes were estimated to be 10.9% and 35.7%, respectively [1]. The middle-aged and elderly Chinese are at even higher risk of diabetes and prediabetes than younger population [1, 2]. Diabetes is associated with various vascular and non-vascular complications and significantly increased risk of all-cause mortality [3]. In the light of the latest Chinese Census data, 13.31% of the population has aged over 60 years with 8.91% aged over 65 years. Moreover, age group 40–49 years and 50–59 years account for 17.28% and 12.01% of the population, respectively [4]. With the increasing trend of aging in China, the disease burdens of diabetes and its related complications such as cardiovascular diseases (CVD) will be further worsen.

Insulin resistance and impaired β-cell function are the important pathological basis for the deterioration of glucose metabolism and type 2 diabetes (T2DM) [5]. Results from WhiteHall II population-based prospective study showed that in South Asian, abnormal glucose tolerance happened 3–5 years after insulin sensitivity significantly declined, whereas β-cell function remained unchanged in this period, indicating that insulin resistance maybe the determinant component in the conversion from normal glucose tolerance to prediabetes [6]. However, the recent study by Gastaldelli et al. [7] showed that the progressive deterioration in β-cell function begins in “normal” glucose tolerant subjects, and is associated with a progressive increase in free fat acid (FFA) and fasting adipose tissue insulin resistance (Adipo-IR). Thus, the determinant factor for the conversion from normal glucose tolerance (NGT) to prediabetes need to be discussed.

The relative contribution of insulin resistance and impaired β-cell function in the development of T2DM is controversy as well. Insulin resistance and impaired β-cell function co-existed in prediabetes in Chinese [8], Korean [9], Japanese [10] and many other population. Several studies have suggested that the fast development of insulin resistance over time leading to the failure of a compensatory increase in insulin is the driven cause of the conversion from prediabetes to T2DM [11–13], while others regarded the deterioration of β-cell function over time as the primary pathology [14–16]. This disparity may have been resulted from ethnicity, as previous study has reported ethic difference in the rate of deterioration to T2DM, with higher conversion rate observed in South Asian and African origin than those of European origin. However, even both conducted in the Chinese population, one study suggested the dominant factor of this conversion to be the deterioration of β-cell function [15], when the other reported the failure to compensate for the deterioration of insulin resistance to be the primary factor [13]. Thus, the dominant factor in prediabetes and T2DM remains to be further studied, especially in the high-risk Chinese population, namely, the middle-aged and the elderly.

Facing this growing epidemic of T2DM in China and the high prevalence in the middle-aged and the elderly, this study aimed to examine the determinant of developing prediabetes and diabetes in the middle-aged and elderly Chinese population, and our study will provide valuable information for prevention and treatment of abnormal glucose metabolism and T2DM.

## Materials and methods

### Study population

This cross-sectional study was conducted in an administrative village of Lishui city, Zhejiang province from May 16 to June 15, 2017. All 523 individuals aged 50 and over in the village were invited to participate. Among them, 11 refused to participant, 3 had no complete information of postprandial glucose or C-peptide, and finally 509 were included in this analysis. This study was approved by the Ethics Committee of Lishui Municipal Central Hospital (No. 2016-42) and was conducted in accordance with the principles of the Helsinki Declaration II. Written consent was obtained from all participants.

### Clinical assessment

Clinical assessment was conducted in Lishui Hospital of Zhejiang University. All subjects were interviewed by trained research staffs to complete questionnaires pertaining to demographic and socioeconomic status, smoking and drinking status, as well as diabetes history. All participants had a clinical examination, include weight, height, waist and blood pressure. Weight was measured to the nearest 0.1 kg with a standard process, and individuals were asked to wear only light underwear and empty the bladder. Body height, and waist circumference were measured to the nearest 0.1 cm using a flexible anthropometer. Waist circumference (WC) was measured at the midpoint between the lower border of the rib cage and the iliac crest. Blood pressure (BP) was measured in triplicate after 10-min rest with Omron electronic sphygmomanometers; the average of the blood pressure measurements was used for the analysis. All the participants also received a 2-h 75 g oral glucose tolerance test (OGTT) with measurement of plasma glucose, insulin, and FFA concentrations at 0 min and 120 min after glucose ingestion.

### Biochemical analysis

Venous blood samples were obtained after an overnight fast at least 8 h. Measurements of fasting plasma glucose (FPG), hemoglobin A1c (HbA1c), triglycerides (TG), total cholesterol (TC), low-density lipoprotein cholesterol (LDL-C) and high-density lipoprotein cholesterol (HDL-C) were performed in the hospital’s laboratory by standard laboratory procedures. Plasma insulin and C-peptide concentrations were measured by radioimmunoassay using specific kits (DPC, Los Angeles, Calif., USA) and plasma FFA were measured spectrophotometrically (Wako, Neuss, Germany). All the biochemical analysis was done in Lishui Hospital of Zhejiang University.

### Definition and calculation

NGT was defined as having fasting blood glucose level below 6.1 mmol/L and postprandial blood glucose level below 7.8 mmol/L and having no history of diabetes. Prediabetes was defined as having no history of diabetes but with fasting blood glucose level ranging between 6.1 and 6.9 mmol/L or postprandial blood glucose level between 7.8 and 11.0 mmol/L. T2DM was defined as having either a history of T2DM or taking hypoglycemic agents or having fasting blood glucose level over 7.0 mmol/L or postprandial blood glucose level over 11.1 mmol/L [17].

Body mass index (BMI) was calculated as weight in kilograms divided by height in meters squared. Waist to hip ratio (WHR) was calculated as waist circumference divided by hip circumference. The homoeostasis model assessment of insulin resistance (HOMA-IR) and β-cell function (HOMA-β) were calculated as previously described [18]. Adipo-IR was calculated as fasting plasma FFA (mmol/L) × fasting plasma insulin (μU/mL) concentrations. C-peptide to glucose ratio was calculated in the fasting (FCPRI) and postprandial (PCPRI) status.

### Statistical analysis

All statistical analyses were conducted using SPSS software (version 23.0 for windows purchased by Capital Medical University, China, SPSS Inc., Chicago, IL). All variables were analyzed for normal distribution using skewness and kurtosis test. Data were expressed as mean ± standard deviation (SD) for continuous variables with normal distribution, median and interquartile range for skewed variables, and number and percentage for categorical variables, respectively. Comparisons of means, medians and frequencies were done with analysis of variance (ANOVA), Kruskal–Wallis and Chi squared tests, respectively. Bonferroni post hoc test was used for multiple pairwise comparisons among three glucose tolerance groups. Association of insulin resistance and β-cell function with prediabetes or T2DM was evaluated by multivariate logistic regression analysis, in which sex, age, BMI, drinking status, LDL-C and HbA1c were adjusted. All tests were two-sided, and a value of *P* < 0.05 was considered significant.

## Results

### General characteristics of study population

The 509 participants included in this study had a mean age of 60.2 (ranged 49–75 years), and with 236 (46.4%) males and 273 (53.6%) females, among which 263 (51.7%) had NGT, 161 (31.6%) had prediabetes and 85 (16.7%) had T2DM. Compared to individuals with NGT, patients with prediabetes or T2DM were older and more likely to be current drinker, had higher level of adiposity indices (waist circumference, BMI, WHR, calf girth and neck circumference), less favorable metabolic traits (higher level of LDL-C, TG, TC, HbA1c, FPG, 2 h-PG, SBP and DBP) and worse status of insulin resistance (HOMA-IR and Adipo-IR) and β-cell function (HOMA-β, FCPRI and PCPRI) (Table 1). Furthermore, with the stage of abnormal glucose metabolism advancing, the levels of insulin resistance and β-cell function deteriorated (*P*_*trend*_ < 0.05 for all indices except HOMA-β) (Table 1).

**Table 1:** General characteristics of study population by glucose tolerance

Variables	NGT n = 263	Prediabetes n = 161	T2DM n = 85	*P*	*P*_*trend*_
Age (years)	59.4 ± 6.6	60.4 ± 7.4	62.7 ± 6.1^a, b^	0.001	< 0.001
BMI (kg/m^2^)	22.2 ± 2.8	23.4 ± 2.9^a^	23.7 ± 2.9^a^	< 0.001	< 0.001
WHR	0.89 ± 0.06	0.92 ± 0.10^a^	0.93 ± 0.06^a^	< 0.001	< 0.001
Female (n, %)	143 (54.4)	78 (48.4)	52 (61.2)	0.154	0.153
Education (n, %)				0.135	0.124
Primary school and below	157 (59.7)	91 (56.5)	62 (71.9)		
Middle school	76 (28.9)	53 (32.9)	17 (20.0)		
High school and above	30 (11.4)	17 (10.6)	6 (7.1)		
Smoking status (n, %)				0.163	0.158
Non-smoker	165 (62.7)	93 (57.8)	55 (64.7)		
Ever-smoker	28 (10.7)	29 (18.0)	1 (16.5)		
Current-smoker	70 (26.6)	39 (24.2)	16 (18.8)		
Drinking status (n, %)				0.029	0.026
Non-drinker	118 (44.9)	66 (41.0)	49 (57.7)		
Ever-drinker	17 (6.4)	4 (2.5)	4 (4.7)		
Current-drinker	128 (48.7)	91 (56.5)	32 (37.6)		
LDL-C (mmol/L)	2.50 ± 0.67	2.73 ± 0.72^a^	2.64 ± 0.76	0.003	0.015
HDL-C (mmol/L)	1.34 ± 0.32	1.31 ± 0.33	1.34 ± 0.38	0.740	0.785
TG (mmol/L)	1.42 ± 0.90	1.78 ± 1.14^a^	2.01 ± 1.50^a^	< 0.001	< 0.001
TC (mmol/L)	4.77 ± 0.85	5.14 ± 0.94^a^	5.20 ± 1.00^a^	< 0.001	< 0.001
HbA1c (%)	5.52 ± 0.34	5.65 ± 0.32	6.72 ± 0.71^a, b^	< 0.001	< 0.001
FPG (mmol/L)	5.24 ± 0.37	5.55 ± 0.49	7.43 ± 0.57^a, b^	< 0.001	< 0.001
2 h-PG (mmol/L)	6.26 ± 1.05	8.90 ± 1.20^a^	13.92 ± 1.38^a, b^	< 0.001	< 0.001
SBP (mmHg)	120.6 ± 15.8	127.2 ± 13.2^a^	130.7 ± 16.2^a^	< 0.001	< 0.001
DBP (mmHg)	71.1 ± 9.2	74.5 ± 7.9^a^	75.4 ± 8.4^a^	< 0.001	< 0.001
Adipo-IR					
Q1 (≤ 1.52)	92 (35.0)	23 (14.3)	11 (12.9)	< 0.001	< 0.001
Q2 (1.53–2.37)	68 (25.9)	40 (24.8)	13 (15.3)		
Q3 (2.38–3.70)	68 (25.9)	42 (26.1)	20 (23.5)		
Q4 (≥ 3.71)	35 (13.3)	56 (34.8)	41 (48.2)		
HOMA-IR					
Q1 (≤ 0.87)	82 (31.2)	28 (17.4)	11 (12.9)	< 0.001	< 0.001
Q2 (0.88–1.29)	76 (28.9)	38 (23.6)	8 (9.4)		
Q3 (1.30–1.91)	66 (25.1)	45 (28.0)	23 (27.1)		
Q4 (≥ 1.92)	39 (14.8)	50 (31.0)	43 (50.6)		
HOMA-β					
Q1 (≤ 40.12)	59 (22.4)	36 (22.3)	43 (50.6)	< 0.001	0.135
Q2 (40.13–56.04)	76 (28.9)	33 (20.5)	17 (20.0)		
Q3 (56.05–80.23)	69 (26.3)	45 (28.0)	10 (11.8)		
Q4 (≥ 80.24)	59 (22.4)	47 (29.2)	15 (17.6)		
Fasting CPRI					
Q1 (≤ 1.16)	70 (26.6)	30 (18.6)	28 (32.9)	0.005	0.005
Q2 (1.17–1.48)	77 (29.3)	32 (19.9)	25 (29.4)		
Q3 (1.49–1.92)	62 (23.6)	48 (29.8)	14 (16.5)		
Q4 (≥ 1.93)	54 (20.5)	51 (31.7)	18 (21.2)		
Postprandial CPRI					
Q1 (≤ 4.41)	54 (20.5)	31 (19.3)	36 (42.4)	< 0.001	< 0.001
Q2 (4.42–5.53)	62 (23.6)	48 (29.8)	10 (11.8)		
Q3 (5.54–7.08)	69 (26.2)	44 (27.3)	7 (8.3)		
Q4 (≥ 7.09)	78 (29.7)	38 (23.6)	3 (3.5)		

Data are expressed as mean ± SD or number (percentage). *P:* from analysis of variance; *P*_trend_: from test for linearity. Differences among three glucose tolerance groups were analyzed by Bonferroni-Dunn post hoc test

^a^Statistically significant vs. NGT

^b^Statistically significant vs. prediabetes

### Insulin resistance but not β-cell function is associated with prediabetes

To analyze the influence of insulin resistance and β-cell function on prediabetes and to better explain the results, we excluded those with T2DM and divided the subjects into 4 groups based on the quartiles of indices of insulin resistance and β-cell function.

Both Adipo-IR and HOMA-IR were higher in prediabetes than NGT. In multivariate model, subjects in the second, third and fourth quartiles of Adipo-IR being inflicted with prediabetes was 2.35-, 2.47- and 6.40-fold the risk of subjects in the first quartile, similarly, HOMA-IR in quartile 3 [odds ratio (OR), 95% CI 2.00 (1.13–3.54)] and quartile 4 [OR, 95% CI 3.76 (2.06–6.84)] were also associated with increased risk of prediabetes compared to the first quartile of HOMA-IR, respectively. In confounders adjusted models, being in the quartile 2–4 of Adipo-IR (risking 1.78, 1.74 and 6.03-fold the risk, respectively), and quartile 4 of HOMA-IR (rising 2.01-fold the risk) remained as risk factors of prediabetes while quartile 3 of HOMA-IR no longer showed any association with prediabetes.

In primary analysis, distribution of the three indices of β-cell function was statistically significant. Multivariate model results showed that only FCPRI in quartile 3 and quartile 4 were associated with 1.81- and 2.20-fold the risk of prediabetes compared to the first quartile of FCPRI. However, no association between FCPRI and prediabetes was found after confounders adjusted (Table 2).

**Table 2:** Association of indices of insulin resistance and β-cell function to glucose tolerance status

Variables	Prediabetes vs. NGT		T2DM vs. prediabetes	
	Crude OR (95% CI)	Adjusted OR (95% CI)	Crude OR (95% CI)	Adjusted OR (95% CI)
Adipo-IR				
Q1 (≤ 1.52)	Ref.	Ref.	Ref.	Ref.
Q2 (1.53–2.37)	*2.35 (1.29–4.29)*	*2.78 (1.44–5.38)*	0.68 (0.26–1.76)	0.71 (0.21–2.38)
Q3 (2.38–3.70)	*2.47 (1.36–4.49)*	*2.74 (1.37–5.46)*	1.00 (0.41–2.44)	0.84 (0.26–2.78)
Q4 (≥ 3.71)	*6.40 (3.43–11.92)*	*7.03 (3.23–15.32)*	1.53 (0.67–3.49)	1.74 (0.51–5.96)
HOMA-IR				
Q1 (≤ 0.87)	Ref.	Ref.	Ref.	Ref.
Q2 (0.88–1.29)	1.46 (0.82–2.61)	1.31 (0.68–2.50)	0.54 (0.19–1.51)	0.32 (0.09–1.09)
Q3 (1.30–1.91)	*2.00 (1.13–3.54)*	1.77 (0.87–3.60)	1.30 (0.55–3.07)	0.85 (0.28–2.61)
Q4 (≥ 1.92)	*3.76 (2.06–6.84)*	*3.01 (1.40–6.48)*	2.19 (0.98–4.91)	0.74 (0.22–2.46)
HOMA-β				
Q1 (≤ 40.12)	Ref.	Ref.	Ref.	Ref.
Q2 (40.13–56.04)	0.71 (0.40–1.27)	0.62 (0.33–1.19)	*0.43 (0.21–0.90)*	0.45 (0.17–1.22)
Q3 (56.05–80.23)	1.07 (0.61–1.87)	0.90 (0.46–1.76)	*0.19 (0.08–0.42)*	*0.11 (0.04–0.34)*
Q4 (≥ 80.24)	1.31 (0.74–2.30)	0.87 (0.41–1.86)	*0.27 (0.13–0.56)*	*0.22 (0.08–0.66)*
Fasting CPRI				
Q1 (≤ 1.16)	Ref.	Ref.	Ref.	Ref.
Q2 (1.17–1.48)	0.97 (0.54–1.76)	0.73 (0.39–1.38)	0.84 (0.40–1.74)	1.00 (0.38–2.64)
Q3 (1.49–1.92)	*1.81 (1.02–3.19)*	1.25 (0.65–2.41)	*0.31 (0.14–0.69)*	*0.24 (0.07–0.75)*
Q4 (≥ 1.93)	*2.20 (1.24–3.91)*	1.27 (0.61–2.66)	*0.38 (0.18–0.80)*	*0.26 (0.08–0.83)*
Postprandial CPRI				
Q1 (≤ 4.41)	Ref.	Ref.	Ref.	Ref.
Q2 (4.42–5.53)	1.35 (0.76–2.41)	1.14 (0.60–2.13)	*0.18 (0.08–0.41)*	*0.16 (0.06–0.46)*
Q3 (5.54–7.08)	1.11 (0.62–1.99)	0.78 (0.41–1.48)	*0.14 (0.05–0.35)*	*0.22 (0.08–0.63)*
Q4 (≥ 7.09)	0.85 (0.47–1.53)	0.55 (0.28–1.07)	*0.07 (0.02–0.24)*	*0.08 (0.02–0.31)*

*P* and OR: from multivariate logistic regression analysis. The adjusted model included sex, age, BMI, drinking status, LDL-C and HbA1c

Statistically significance values are in italics (*p* < 0.05)

### β-Cell function but not insulin resistance is associated with T2DM

To examine whether insulin resistance and β-cell function were differentially associated with T2DM, we included only the subjects with prediabetes and T2DM. As shown in Table 2, neither HOMA-IR nor Adipo-IR were associated with T2DM in unadjusted model and confounders adjusted model.

For the three indices of β-cell function, compared to those counterparts in the first quartile, being in the second, third and fourth quartiles of HOMA-β showed 0.43-, 0.19- and 0.27-fold the risk of T2DM, being in the third and fourth quartile of FCPRI showed 0.31- and 0.38-fold the risk, and being in the quartile 2–4 of PCPRI showed 0.18-, 0.14- and 0.07-fold the risk. Being in the quartile 3 and 4 of HOMA-β (reducing 0.89- and 0.78-fold the risk), quartile 3 and 4 of FCPRI (reducing 0.76- and 0.74-fold the risk), and quartile 2–4 of PCPRI (reducing 0.84-, 0.78- and 0.92-fold the risk, respectively) remained as protective factors of T2DM, after adjusting for sex, age, BMI, drinking status, LDL-C and HbA1c (Table 2).

## Discussion

In this study, although we showed that from normal glucose tolerance to prediabetes and diabetes, the insulin resistance (HOMA-IR and Adipo-IR) and β-cell function (FCPRI, PCPRI) were getting deterioration (*P*_*trend*_ < 0.05 for all indices), only insulin resistance was differentially distributed between NGT and prediabetes, and β-cell function was differentially distributed between prediabetes and diabetes, indicating that insulin resistance and β-cell dysfunction were the determinant of developing prediabetes and diabetes, respectively.

Insulin resistance is a well-established major cause for abnormal glucose metabolism. Some studies have observed an increased insulin resistance in impaired glucose tolerance (IGT) and T2DM patients [19, 20]. When HOMA-IR as a validated index for insulin resistance has been widely studied, there are only two reports on adipose tissue insulin resistance in the natural history of T2DM, one in obese adolescents and the other in the population of South Texas [7, 21]. Adipose tissue is an endocrine organ able to influence both systemic inflammation and metabolic homoeostasis. One action of the insulin on adipose tissue is suppressing triglyceride hydrolysis and release of FFA and glycerol into the circulation [22]. The lipolysis suppression effect was weakened in the case of adipose tissue insulin resistance, resulting in excess FFA delivery to other tissues and thus contributing to ectopic fat deposition [23]. Moreover, strengthened lipolysis induced by adipose tissue insulin resistance may be a key mediator in the early stages of metabolic derangements, as excess FFA has been shown to impair muscle insulin signaling, promote hepatic gluconeogenesis and impair glucose-stimulated insulin response, all of which is detrimental to whole-body insulin sensitivity and metabolism [24–26]. Recently, Adipo-IR, a simple index calculated as fasting insulin × fasting FFA, has been validated against the gold standard measurement method (that is, measurement of the rate of appearance (Ra) of glycerol during hyperinsulinemic-euglycemic clamp conditions and the multistep pancreatic clamp technique), and the index turned out to be a good predictor of a gold standard measure of adipose tissue insulin sensitivity [27, 28]. In consistence with the study of Gastaldelli et al., we found that with the stage of abnormal glucose metabolism advancing, the level of Adipo-IR deteriorated. Furthermore, Adipo-IR had stronger association with prediabetes than HOMA-IR. One plausible explanation might be that adipose tissue insulin resistance can promote ectopic fat deposition, resulting in more fat tissue thus causing elevated adipose tissue insulin resistance while promoting insulin resistance in the muscle and liver, all of which plays a critical role in the pathological process of abnormal glucose metabolism.

Although T2DM has been considered merely a deficit of insulin action over the past few decades, diminished β-cell function is now widely recognized as a core feature of T2DM after Butler et al. and other groups reported β-cell deficit with reduced β-cell mass in T2DM patients [29, 30]. Our study showed that higher level of PCPRI is associated with significant lower risk of T2DM in prediabetes individuals. Insulin secretion increases in the postprandial state as a response to the stimulation of elevated glucose level and incretin [31]. This makes PCPRI a more reliable indicator of the maximal insulin secretory capacity compared to FCPRI and HOMA-β, especially in patients with T2DM. Furthermore, previous studies have reported that PCPRI showed strongest correlation to β-cell area compared to FCPRI and HOMA-β, in which they also described the observation that β-cell area was significantly different between IGT and T2DM (*P* < 0.01) and between NGT and T2DM (*P *< 0.01) [32]. Considering the close relation between β-cell mass and PCPRI, this observation might be an explanation for the strong negative association with T2DM in both NGT and prediabetes. The clinical potential of CPRI in T2DM has been explored in previous studies. Peacock and Tattersall reported almost three decades ago that FCPRI can help predict the response to insulin in patients on insulin therapy [33]. Recent researches have expended the utility of FCPRI to predicting both the future requirements for insulin and achievement of the good glycemic control by insulin [34–36]. Moreover, PCPRI showed better predictive performance than FCPRI. PCPRI has been reported to predict the requirement for multiple daily insulin injections in patients with T2DM and that this index was more useful for predicting treatment strategies such as oral anti-diabetic agents and insulin therapy than other C-peptide indices [37, 38]. All these studies showed that PCPRI has a clinical potential in indicating insulin therapy and achieving target glucose control in T2DM, and the result of ours showed that PCPRI can also be utilized to show the risk of progressing to T2DM in middle-aged and elderly individuals.

The present study has several limitations. First, the cross-sectional design makes it unable to infer causality from the associations observed, although the important role that insulin resistance and β-cell dysfunction played in the pathology of diabetes has been well illustrated in previous studies. Secondly, participants in our study were middle-aged and elderly Chinese adults, so whether these results can be applied in other age groups or other races need to be further validated. Despite these limitations, our study is among the first to explore the characteristics of Adipo-IR and CPRI in the natural history of T2DM in Chinese population, and we showed that Adipo-IR and PCPRI, both of which were simple to calculate, have great clinical potentials for identifying high-risk individuals of prediabetes and T2DM in middle-aged and elderly Chinese adults.

## Conclusions

Our study shows that insulin resistance and β-cell function impairment existed in prediabetes. Moreover, insulin resistance and β-cell function were the main determinant of developing prediabetes and T2DM, respectively. Our study indicates that the status of insulin resistance maybe a key point in the development of NGT to prediabetes, and that in the status of prediabetes, it is of great importance to reserve the β-cell function to prevent further deterioration.

## References

[1] Wang L, Gao P, Zhang M (2017). Prevalence and ethnic pattern of diabetes and prediabetes in China in 2013. JAMA.

[2] Zhao Y, Crimmins EM, Hu P (2016). Prevalence, diagnosis, and management of diabetes mellitus among older Chinese: results from the China health and retirement longitudinal study. Int J Public Health.

[3] Bragg F, Holmes MV, Iona A (2017). Association between diabetes and cause-specific mortality in rural and urban areas of China. JAMA.

[4] Tabulation on the 2010 population census of the People’s Republic of China. http://www.stats.gov.cn/english/Statisticaldata/CensusData/rkpc2010/indexch.htm. Accessed 2 Feb 2019.

[5] Kanaya AM, Herrington D, Vittinghoff E (2014). Understanding the high prevalence of diabetes in US south Asians compared with four racial/ethnic groups: the MASALA and MESA studies. Diabetes Care.

[6] Hulman A, Simmons RK, Brunner EJ (2017). Trajectories of glycaemia, insulin sensitivity and insulin secretion in South Asian and white individuals before diagnosis of type 2 diabetes: a longitudinal analysis from the Whitehall II cohort study. Diabetologia.

[7] Gastaldelli A, Gaggini M, DeFronzo RA (2017). Role of adipose tissue insulin resistance in the natural history of type 2 diabetes: results from the San Antonio metabolism study. Diabetes.

[8] Yue Y, He H, Yang XJ (2016). Pancreatic beta-cell functions measured by continuous glucose monitoring in Han Chinese with varied degree of glucose tolerance. Sichuan da xue xue bao Yi xue ban = J Sichuan Univ Med Sci Ed.

[9] Rhee SY, Kim JY, Chon S (2010). The changes in early phase insulin secretion in newly diagnosed, drug naive korean prediabetes subjects. Korean Diabetes J.

[10] Onishi Y, Hayashi T, Sato KK (2010). Fasting tests of insulin secretion and sensitivity predict future prediabetes in Japanese with normal glucose tolerance. J Diabetes Investig.

[11] Festa A, Williams K, D’Agostino R (2006). The natural course of beta-cell function in nondiabetic and diabetic individuals: the insulin resistance atherosclerosis study. Diabetes.

[12] Tabak AG, Jokela M, Akbaraly TN (2009). Trajectories of glycaemia, insulin sensitivity, and insulin secretion before diagnosis of type 2 diabetes: an analysis from the Whitehall II study. Lancet (London, England).

[13] Liu J, Wu YY, Huang XM (2014). Ageing and type 2 diabetes in an elderly Chinese population: the role of insulin resistance and beta cell dysfunction. Eur Rev Med Pharmacol Sci.

[14] Cnop M, Vidal J, Hull RL (2007). Progressive loss of beta-cell function leads to worsening glucose tolerance in first-degree relatives of subjects with type 2 diabetes. Diabetes Care.

[15] Qian L, Xu L, Wang X (2009). Early insulin secretion failure leads to diabetes in Chinese subjects with impaired glucose regulation. Diabetes/Metab Res Rev.

[16] Morimoto A, Tatsumi Y, Deura K (2013). Impact of impaired insulin secretion and insulin resistance on the incidence of type 2 diabetes mellitus in a Japanese population: the Saku study. Diabetologia.

[17] Alberti KG, Zimmet PZ (1998). Definition, diagnosis and classification of diabetes mellitus and its complications. Part 1: diagnosis and classification of diabetes mellitus provisional report of a WHO consultation. Diabet Med.

[18] Matsuda M, DeFronzo RA (1999). Insulin sensitivity indices obtained from oral glucose tolerance testing: comparison with the euglycemic insulin clamp. Diabetes Care.

[19] Abu-Farha M, Behbehani K, Elkum N (2014). Comprehensive analysis of circulating adipokines and hsCRP association with cardiovascular disease risk factors and metabolic syndrome in Arabs. Cardiovasc Diabetol.

[20] Barchetta I, Angelico F, Del Ben M (2016). Phenotypical heterogeneity linked to adipose tissue dysfunction in patients with type 2 diabetes. Clin Sci.

[21] Hershkop K, Besor O, Santoro N (2016). Adipose insulin resistance in obese adolescents across the spectrum of glucose tolerance. J Clin Endocrinol Metab.

[22] Saponaro C, Gaggini M, Carli F (2015). The subtle balance between lipolysis and lipogenesis: a critical point in metabolic homeostasis. Nutrients.

[23] Ferrannini E, Barrett EJ, Bevilacqua S (1983). Effect of fatty acids on glucose production and utilization in man. J Clin Investig.

[24] Mundi MS, Koutsari C, Jensen MD (2014). Effects of increased free fatty acid availability on adipose tissue fatty acid storage in men. J Clin Endocrinol Metab.

[25] Gyllenhammer LE, Alderete TL, Toledo-Corral CM (2005). Saturation of subcutaneous adipose tissue expansion and accumulation of ectopic fat associated with metabolic dysfunction during late and post-pubertal growth. Int J Obes.

[26] Morelli M, Gaggini M, Daniele G (2013). Ectopic fat: the true culprit linking obesity and cardiovascular disease?. Thromb Haemost.

[27] Ter Horst KW, van Galen KA, Gilijamse PW (2005). Methods for quantifying adipose tissue insulin resistance in overweight/obese humans. Int J Obes.

[28] Sondergaard E, Espinosa De Ycaza AE, Morgan-Bathke M (2017). How to measure adipose tissue insulin sensitivity. J Clin Endocrinol Metab.

[29] Butler AE, Janson J, Bonner-Weir S (2003). Beta-cell deficit and increased beta-cell apoptosis in humans with type 2 diabetes. Diabetes.

[30] Saisho Y (2015). Beta-cell dysfunction: its critical role in prevention and management of type 2 diabetes. World J Diabetes.

[31] Okuno Y, Komada H, Sakaguchi K (2013). Postprandial serum C-peptide to plasma glucose concentration ratio correlates with oral glucose tolerance test- and glucose clamp-based disposition indexes. Metab Clin Exp.

[32] Meier JJ, Menge BA, Breuer TG (2009). Functional assessment of pancreatic beta-cell area in humans. Diabetes.

[33] Peacock I, Tattersall RB (1984). The difficult choice of treatment for poorly controlled maturity onset diabetes: tablets or insulin?. Br Med J.

[34] Funakoshi S, Fujimoto S, Hamasaki A (2011). Utility of indices using C-peptide levels for indication of insulin therapy to achieve good glycemic control in Japanese patients with type 2 diabetes. J Diabetes Investig.

[35] Saisho Y, Kou K, Tanaka K (2011). Postprandial serum C-peptide to plasma glucose ratio as a predictor of subsequent insulin treatment in patients with type 2 diabetes. Endocr J.

[36] Goto A, Takaichi M, Kishimoto M (2010). Body mass index, fasting plasma glucose levels, and C-peptide levels as predictors of the future insulin use in Japanese type 2 diabetic patients. Endocr J.

[37] Fujiwara D, Takahashi K, Suzuki T (2013). Postprandial serum C-peptide value is the optimal index to identify patients with non-obese type 2 diabetes who require multiple daily insulin injection: analysis of C-peptide values before and after short-term intensive insulin therapy. J Diabetes Investig.

[38] Lee EY, Hwang S, Lee SH (2014). Postprandial C-peptide to glucose ratio as a predictor of beta-cell function and its usefulness for staged management of type 2 diabetes. J Diabetes Investig.

